# The Epigenetic Link between Prenatal Adverse Environments and Neurodevelopmental Disorders

**DOI:** 10.3390/genes8030104

**Published:** 2017-03-18

**Authors:** Marija Kundakovic, Ivana Jaric

**Affiliations:** Department of Biological Sciences, Fordham University, 441 E. Fordham Road, Bronx, NY 10458, USA; ijaric@fordham.edu

**Keywords:** epigenetic, DNA methylation, prenatal environment, early life exposures, developmental programming, neurodevelopmental disorders, schizophrenia, depression

## Abstract

Prenatal adverse environments, such as maternal stress, toxicological exposures, and viral infections, can disrupt normal brain development and contribute to neurodevelopmental disorders, including schizophrenia, depression, and autism. Increasing evidence shows that these short- and long-term effects of prenatal exposures on brain structure and function are mediated by epigenetic mechanisms. Animal studies demonstrate that prenatal exposure to stress, toxins, viral mimetics, and drugs induces lasting epigenetic changes in the brain, including genes encoding glucocorticoid receptor (*Nr3c1)* and brain-derived neurotrophic factor (*Bdnf*). These epigenetic changes have been linked to changes in brain gene expression, stress reactivity, and behavior, and often times, these effects are shown to be dependent on the gestational window of exposure, sex, and exposure level. Although evidence from human studies is more limited, gestational exposure to environmental risks in humans is associated with epigenetic changes in peripheral tissues, and future studies are required to understand whether we can use peripheral biomarkers to predict neurobehavioral outcomes. An extensive research effort combining well-designed human and animal studies, with comprehensive epigenomic analyses of peripheral and brain tissues over time, will be necessary to improve our understanding of the epigenetic basis of neurodevelopmental disorders.

## 1. Prenatal Adversity and Neurodevelopmental Disorders

It is now well-established that adverse prenatal environments, such as exposure to maternal stress, viral infections, drugs, and toxins, can disrupt normal brain development and have lasting consequences for the structure and function of the brain [1,2,3,4,5]. Moreover, prenatal adversity can significantly increase the risk of developing mental disease in later life, including schizophrenia, depression, anxiety, and autism, among others [1,6]. Schizophrenia (SCZ) is, for instance, widely considered to be a neurodevelopmental disorder [7,8] resulting from complex interactions between genetic and environmental risk factors. SCZ runs in families [9], but the concordance rate of monozygotic twins is only about 50%, showing that environmental factors play an important role, likely by influencing fetal brain development [10]. The increased risk of SCZ has been linked to several in utero exposures, including severe food restriction (Dutch Famine Study), exposure to viral infections (e.g., influenza, herpes simplex), stress (in male offspring)**,** and hypoxia associated with gestational and birth complications [11,12].

Importantly, these prenatal risk factors per se do not seem to be specific to SCZ, although the window of exposure may play a particularly important role. As an example, famine exposure occurring during the periconceptional period was associated with an increased risk of SCZ [13], whereas the same exposure during the second and third trimesters of in utero development was linked to affective disorders in offspring [14,15]. Interestingly, maternal stress during pregnancy has also been associated with the increased risk of the development of many neuropsychiatric disorders in the offspring besides SCZ, including depression, autism, and anxiety [1]. In addition, attention deficit hyperactivity disorder (ADHD) has been linked to maternal stress and a poor maternal diet during pregnancy as well as to the prenatal exposure to recreational drugs (e.g., nicotine and alcohol) and toxicants (e.g., polychlorinated biphenyls and fungicide hexachlorobenzene) [16].

Currently, it is not understood why and how a single environmental risk factor would be able to initiate different psychiatric outcomes or how different factors can lead to the same disorder. As previously stated, it seems plausible that the timing of exposure plays a significant role. In addition, complex interactions of an individual’s genetic make-up, sex, and the life-long environment are likely to determine the final outcome. It is certainly important to understand biological mechanisms through which prenatal environmental exposures can affect brain development and predispose an individual to mental disease later in life. An increasing number of studies have shown that psychiatric disorders such as SCZ involve widespread epigenetic dysregulation [17,18], the origin of which was recently traced back to the development [19,20]. Logically, epigenetic mechanisms have emerged as a plausible biological substrate through which prenatal environmental exposures can disrupt normal brain development and induce lasting effects on brain function and behavior [1,16,21,22], and this will be discussed in the following section.

## 2. Epigenetic Mechanisms and Prenatal Programming of Brain Function

Epigenetic mechanisms, such as DNA methylation and histone modifications, regulate gene expression and are essential for normal development and cellular differentiation, including the differentiation of brain cells [23,24,25]. DNA methylation is the best studied epigenetic mark; it occurs at position 5 of cytosine, primarily in the context of cytosine–guanine (CpG) dinucleotides, and is catalyzed by DNA methyltransferases (DNMTs). Cytosine methylation within gene promoters has been typically associated with transcriptional repression, acting via two possible mechanisms: (1) by directly impeding the binding of transcription factors; or (2) by locally inducing a repressive chromatin structure that is nonpermissive to transcription [26]. However, the relationship between DNA methylation and the regulation of gene expression has been found to be complex, as highlighted by more recent studies. First, in addition to CpG methylation, a significant portion of methylation is found to be positioned at non-CpG sites, particularly in neuronal cells [27]. Second, the interrelation between cytosine methylation and gene expression is more complex than previously thought, with many other genomic regions, including gene bodies, being subject to DNA methylation, resulting in the repression or activation of genes, depending on the context [26,28,29,30,31]. Third, additional methylation-related cytosine modifications have been described, with a growing interest in hydroxymethylation, which involves the ten-eleven translocation (Tet) proteins and is suggested to be an intermediate for the removal of methylated cytosines [27,32]. Finally, it is important to mention the emerging roles of DNA methylation beyond the regulation of gene expression, including its effect on alternative splicing [33] and a higher order chromatin structure [34]. These new findings highlight the complexity of the DNA methylome and require the careful interpretation of epigenetic findings, including those associated with prenatal environmental exposures.

Postranslational modifications of histones are even more diverse and complicated than DNA methylation, with more than 100 different modifications currently being described as contributing to either active of repressive chromatin states, depending on the context [35]. For instance, it is known that H3K4me3 mark is primarily associated with active gene promoters, H3K27me3 is associated with repressed regulatory regions [36], whereas acetylation marks such as H3K27ac are associated with active promoter and enhancer regions [37,38]. Importantly, it is known that DNA methylation and histone modifications can also interact to regulate chromatin structure and gene expression [39].

**Figure 1:**
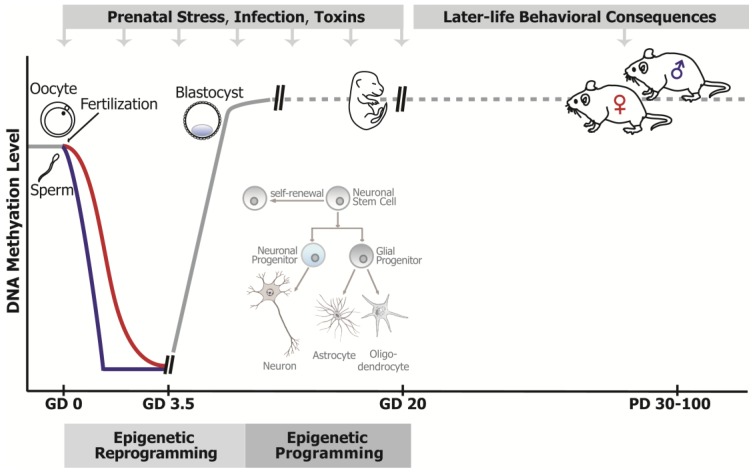
The epigenome as a substrate for the lasting effects of prenatal stressors on brain function and behavior. The epigenome is particularly vulnerable to disruption by environmental agents during prenatal development, when an extensive reprogramming and programming of epigenetic modifications takes place. The post-fertilization “epigenetic reprogramming” (zygote to blastocyte stage) includes the almost complete erasure of DNA methylation in both the paternal (blue line) and the maternal (red line) genome, which is then re-established (solid gray line), leading to differential DNA methylation and gene expression patterns in the first cell lineages. In the later stages of development, epigenetic marks are less dynamic (dashed gray line), but still actively participate in gene expression programming, relevant for later stages of cellular differentiation (“epigenetic programming”). As an example, during the differentiation of brain cells (see picture inset), DNA methylation and histone modifications are involved in the gene expression programming that differentiates neuronal stem cells into neuronal and glial progenitors, and further into more specialized neuronal and glial cells (astrocytes and oligodendrocytes). Hence, prenatal exposure to environmental factors that affect the epigenome (stress, infection, toxins) can disrupt gene expression programming in the embryo/fetus, resulting in developmental deficits, including abnormal brain development that can lead to later-life behavioral disorders. Importantly, the epigenome is also dynamic in mature, postmitotic neurons (depicted as a dashed gray line postnatally), so long-term behavioral abnormalities may also result from the improper developmental programming of the brain’s epigenetic machinery that continues to be used by mature neurons.

While one of the most important characteristics of epigenetic marks is that they are dynamic and responsive to environmental cues throughout life, prenatal development represents the most vulnerable period for epigenetic disruption (Figure 1) [40]. The epigenome is highly susceptible to environmental exposures (e.g., maternal stress, toxins, drugs, pollutants) during early prenatal development, when extensive epigenetic reprogramming (resetting the human epigenome for naive pluripotency) and epigenetic programming (epigenetic alterations driving cellular differentiation) take place, in order to establish cell- and tissue-specific gene expression. Pre-implantation epigenetic reprogramming, occurring from the zygote through the blastocyst stage (Figure 1), involves the genome-wide erasure and remodeling of DNA methylation and histone modifications [41]. These events are thought to be required for the establishment of the zygote’s totipotency, initiation of embryonic gene expression, and early lineage development in the embryo [41]. It is therefore obvious that interference with epigenetic reprogramming during early embryogenesis can significantly impact early gene programming in the developing embryo [42,43].

Although changes in the epigenome are not so extreme during later developmental stages, epigenetic modifications also actively participate in controlling gene expression in later lineage commitment (Figure 1). In terms of neurodevelopment, there is an epigenetic “programming” of stem cells into neuronal precursors, followed by further differentiation into specific neurons or glia cells (Figure 1) [23,24,25]. During neurogenesis, neural precursor cells express factors that promote the transcription of neuronal genes, whereas some glia-specific genes are repressed by DNA methylation [25]. On the other hand, GFAP is a glial protein marker specifically expressed by astrocytes and not found in neurons. In mice, the *Gfap* promoter has to be demethylated to allow *Gfap* gene expression, and hence, astrocyte differentiation; all non-expressing cells, including neurons, show high levels of DNA methylation within this region [44]. In vivo evidence was provided using conditional knockout mice with a specific deletion of DNA methyltransferase 1 (*Dnmt1*) gene in neural progenitor cells of the developing brain [45]. Brains of these mice show reduced numbers of neurons and precocious astroglial differentiation, confirming that the neuro-to-gliogenic switch involves DNA methylation-dependent mechanisms.

Therefore, the epigenome represents the plausible molecular substrate through which environmental exposures can change the patterns of gene expression associated with normal neurodevelopment and significantly impact brain structure and function. However, the question is how epigenetic mechanisms may mediate long-term effects of prenatal stressors on brain function and behavior observed later in life (Figure 1). This is particularly challenging to understand with regards to mental disorders such as SCZ, which present in late adolescence or early adulthood and are known to be associated with globally dysregulated DNA methylation patterns in the brain [17,18,19,20]. Multiple prenatal environmental exposures associated with an increased risk of psychiatric disorders (e.g., stress, toxicants, drugs) have been shown to impact DNA methylation levels and gene expression in the brain, both in the short- and long-term (Table 1). One possible mechanism for the long-term effects of prenatal environments is that, once established, aberrant DNA methylation patterns can be passed from one cell generation to another (in immature, still dividing cells), or they are simply stably maintained into adulthood (in mature, postmitotic neurons), thus providing the mechanism through which the early life environment can exert long-lasting effects on gene expression and phenotype [46]. However, as we will show with examples in this article, this is not the only possible mechanism that could underlie long-term effects. In fact, increasing evidence shows that DNA methylation in mature, postmitotic neurons is dynamic and actively involved in neuronal gene regulation important for neuroplasticity [47]. Therefore, an alternative mechanism could be that prenatal environmental agents induce improper programming of the brain’s epigenetic machinery (e.g., DNMTs, Tet proteins, and histone modifiers). Considering that this machinery continues to be used by mature, postmitotic brain neurons, the disruption of the epigenome, and consequently the disruption of brain function, would be long-lasting, but the phenotype may also be variable, depending on the age and life conditions [48]. Obviously, it would be important to discover the mechanism(s) through which different environmental exposures could “permanently” affect the brain’s epigenetic machinery.

In summary, it seems likely that the entire prenatal period is vulnerable to epigenetic disruption, and that any agent with the ability to affect the epigenome can cause adverse neurodevelopmental effects, the consequences of which may extend into adulthood (Figure 1). We will now review the experimental evidence demonstrating that prenatal environmental exposures can affect the brain epigenome, leading to lasting consequences for later behavior and brain function.

## 3. Experimental Evidence from Animal Studies

Emerging evidence from animal studies shows that maternal exposure to stress, toxicants, viral infections, and drugs, can alter epigenetic gene programming in the brain and contribute to neurodevelopmental and behavioral deficits in the offspring. The available studies are summarized in Table 1 [48,49,50,51,52,53,54,55,56,57,58,59,60,61,62].

### 3.1. Maternal Stress

Maternal stress during pregnancy is associated with an increased risk of several neuropsychiatric disorders in the offspring, including SCZ, depression, anxiety, and autism [1,11,55]. One likely mechanism through which prenatal stress can influence psychiatric risk is through altering the activity of the hypothalamic-pituitary-adrenal (HPA) axis, a key mediator of stress response. The HPA axis is often found to be dysregulated in psychiatric disorders, particularly in patients suffering from depression and anxiety disorders. Mueller and Bale (2008) used a rodent model to examine whether the epigenetic changes within the two genes related to the HPA axis, the glucocorticoid receptor (*Nr3c1*) and the corticotropin-releasing factor (*Crf*) gene, may be involved in the programming of neurobehavioral changes induced by prenatal stress [55]. Interestingly, but not surprisingly, the effects of prenatal stress were shown to largely depend on the timing of stress exposure as well as on the sex of exposed animals. Only male offspring exposed to chronic, variable stress in early gestation (the first gestational week) displayed depressive-like behavior in adulthood, together with an increased HPA response to stress. These changes were further accompanied by the altered expression of genes important for the HPA axis responsiveness, *Nr3c1* and *Crf*, in the hippocampus and the amygdala, respectively. Changes in *Nr3c1* and *Crf* DNA methylation were inversely correlated with altered gene expression, providing evidence that an epigenetic mechanism may underlie behavioral and gene expression changes induced by prenatal stress exposure.

Brain-derived neurotrophic factor (*Bdnf*) is another candidate gene for which the epigenetic sensitivity to prenatal stress was examined by multiple studies [51,61,62]. BDNF is crucial for neurodevelopment and synaptic plasticity [63], and its deficiency, including *BDNF* promoter hypermethylation, has been linked to multiple psychiatric disorders that are associated with early-life adversity, including SCZ [64], depression [65,66], bipolar disorder [67], and autism [68]. Therefore, interference with the regulation and expression of *BDNF* provides one of the plausible mechanisms through which early-life adverse environments can disrupt neurodevelopment, leading to lasting consequences for brain plasticity, learning, and behavior.

Accordingly, by using restraint stress during pregnancy from gestational day (GD) 7 to GD21 in mice, Dong et al. [51] demonstrated that prenatal stress results in reduced cortical and hippocampal *Bdnf* mRNA expression, which is accompanied by increased DNA methylation and hydroxymethylation at a *Bdnf* regulatory region. Prenatal stress-induced molecular changes were further associated with hyperactivity and impaired social interaction, drawing a possible link between the epigenetic effects of prenatal stress and neurodevelopmental disorders such as SCZ. In addition, Zheng et al. [62] showed that maternal stress exposure during pregnancy induces anxiety- and depression-like behavior in the offspring, which is associated with transcriptional and epigenetic changes within the *Bdnf* gene. This research group specifically looked in the hippocampus and demonstrated that prenatal stress was associated with reduced *Bdnf* expression as well as increased DNA methylation and decreased histone acetylation (H3K14ac) at specific *Bdnf* promoters. Furthermore, St-Cyr and McGowan [61] examined the effects of prenatal stress in the form of maternal exposure to predator odor over the second half of the pregnancy. They showed that prenatal exposure to predator odor resulted in behavioral changes in the offspring of both sexes. Specifically, in females, these changes were associated with an increased stress response (increased levels of corticosterone in response to stress) as well as the decreased expression of *Bdnf* in the hippocampus, which was further correlated with reduced cytosine methylation within the *Bdnf* exon IV. Collectively, these studies strongly imply that *Bdnf* epigenetic changes induced by prenatal stress may be involved in the programming of neurobehavioral outcomes induced by prenatal stress.

Studies by Dong et al. [51] and Zheng et al. [62] have also shown that gestational stress induces long-term changes in the expression of epigenetic regulators, such as DNA methylation machinery (DNMT1 and TET1) and histone modifiers (histone deacetylases HDAC1 and HDAC2). The enduring effect on epigenetic machinery may provide a possible mechanism through which prenatal stress induces epigenetic changes, suggesting that the changes may be widespread, including hundreds and thousands of genes. It is therefore not surprising that a recent study also reported prenatal stress-induced epigenetic changes within GABAergic genes, which were correlated with SCZ-related behavioral alterations [56]. Interestingly, it was also shown that altered genome-wide DNA methylation patterns in the hippocampus may underlie the gene-by-environment interaction of a serotonin transporter genetic variant and prenatal stress in increasing the risk for psychopathology, particularly in females [60].

### 3.2. Maternal Toxicological Exposures

In humans, maternal exposure to toxicants, such as flame retardants, pesticides, and plastic ingredients, during pregnancy has been linked to an increased risk of neurodevelopmental and behavioral changes in the offspring [69,70,71], and epigenetic mechanisms have been suggested as possible mediators of these neurotoxic effects [40,72]. However, only a few studies have provided experimental evidence that prenatal exposure to toxicants can induce lasting, functionally important epigenetic changes in the brain (Table 1).

Bisphenol A (BPA) is an estrogenic endocrine disruptor that is widely used in the production of plastics [73]. Numerous animal studies have shown that prenatal BPA exposure can affect brain development and induce lasting behavioral changes, and supporting human studies are emerging [71,74,75,76]. Recently, we demonstrated that prenatal exposure to low, environmentally-relevant doses of BPA induces lasting epigenetic disruption in the brain that may contribute to changes in behavior and learning [48,49,50,51,52,53]. We found changes in the DNA methylation and RNA levels of estrogen receptors (ERs) in the cortex and the hypothalamus of the juvenile offspring prenatally exposed to BPA [53] (Table 1). ERs are critical for the brain’s sexual differentiation and are known to play important roles in social and anxiety-like behaviors [77,78,79], which corresponded with BPA-induced behavioral changes in our study [53]. Importantly, we found concordant changes in the expression of epigenetic regulators, DNA methylatransferases (DNMTs), which provided a possible mechanism through which prenatal BPA treatment may induce lasting epigenetic disruption in the brain. With that in mind, BPA-induced epigenetic changes are likely widespread, impacting the expression of numerous genes that may contribute to BPA-induced neurobehavioral consequences.

Indeed, another gene that was found to be affected by BPA is the previously mentioned *Bdnf*. We showed that maternal BPA exposure during pregnancy results in the lasting down-regulation of *Bdnf* gene expression in the offspring’s hippocampus, evident in both juvenile and adult animals, but only in males [48]. Altered *Bdnf* mRNA levels were associated with DNA methylation changes in the regulatory region of the *Bdnf* gene harboring a methylation-sensitive binding site for the transcription factor CREB (cAMP response element-binding protein) [80]. We further showed that those changes were associated with changes in the expression of the DNA methylation machinery at both time points, as well as with memory deficits in young adult male mice [48]. It is important to note that the CpG sites affected in the juvenile and adult animals that were prenatally exposed to BPA were not identical, although they were within the same *Bdnf* promoter region. This suggests that the entire gene region, rather than specific CpG sites, is developmentally “marked” for epigenetic dysregulation by BPA exposure, most likely due to the improper programming of the brain’s DNA methylation machinery (DNMT1 and putative demethylase GADD45b) that continues to be used by mature, postmitotic brain neurons.

*Bdnf* was also found to be epigenetically dysregulated by another toxicological exposure, which is methylmercury [58]. Onishchenko et al. [58] showed that the developmental exposure to methylmercury resulted in reduced BDNF mRNA in the hippocampal dentate gyrus and depression-like behavior in adulthood, both of which can be restored by antidepressant (fluoxetine) treatment. In addition to behavioral and gene expression changes, methylmercury exposure induced a long-lasting repressive chromatin state within the *Bdnf* promoter region, including increased DNA methylation and repressive histone mark H3K27me3, as well as a reduced mark of active chromatin H3 acetylation, strongly implying the role of epigenetic mechanisms in mediating these effects.

In summary, animal studies of prenatal toxicological exposures provide strong evidence that maternal exposure to environmental toxicants can lead to lasting effects on behavior and learning in the offspring, which are, at least in part, mediated by epigenetic mechanisms. Importantly, those changes are often sex-specific and dose-dependent [48,53], thus calling for the careful interpretation of toxicological studies in animals and humans.

### 3.3. Maternal Immune Activation

Maternal exposure to viral infections and the associated immune activation during pregnancy have been linked to various neurobehavioral outcomes in the offspring. In particular, multiple epidemiological studies have established the link between maternal viral infections (such as influenza and herpes simplex) and an increased risk of SCZ and related neurodevelopmental disorders in the offspring [81]. Recent animal studies have explored a possible role of epigenetic mechanisms in mediating long-term neurobehavioral effects of prenatal immune activation [49,54,59].

A well-established mouse model used to study prenatal viral-like immune activation includes a single injection of a viral mimetic polyriboinosinic-polyribocytidylic acid [poly(I:C)], an analogue of double-stranded RNA, to pregnant dams. Basil et al. [49] used this paradigm and exposed mice to the viral analog Poly(I:C) or saline in mid-gestation (GD9) and harvested brain tissue from six week-old offspring. They found that prenatal exposure to Poly(I:C) caused significant global DNA hypomethylation, especially in females, and significant hypomethylation of the promoter region of *Mecp2* in the hypothalamus. While this study demonstrated that prenatal immune activation may be associated with lasting epigenetic changes in the offspring, the authors did not link these changes to any particular functional outcome such as changes in gene expression or behavioral phenotype.

Labouesse et al. [54] focused on Poly(I:C) administration in late gestation (GD17) and the consequent functional impairments within the offspring’s GABAergic neuronal system in the medial prefrontal cortex. They found that prenatal immune activation resulted in reduced mRNA expression of glutamic acid decarboxylase *(Gad)1* and *Gad2*, the two genes that encode GABA-synthesizing enzymes GAD67 and GAD65, respectively. The gene expression changes were accompanied by increased DNA methylation and MeCP2 binding to the *Gad1* regulatory region, and with prenatal immune activation-induced impairments in working memory and social interaction. This study provided important evidence that lasting epigenetic changes within the GABA-relevant genes, such as *Gad1* and *Gad2*, may be an important molecular mechanism linking prenatal infection to GABAergic dysfunction and associated behavioral and cognitive abnormalities in the offspring.

Finally, the most recent study by Richetto et al. [59] considered possible critical windows of vulnerability to infectious insults and examined two developmental windows: mid-gestation (GD9) and late gestation (GD17). The effects of prenatal immune activation on adult behavioral phenotypes were found to be developmental window-specific: both GD9 and GD17 gestational treatments induced social interaction deficits; however, impaired sensorimotor gating was specific to the mid-gestation exposure and impaired spatial memory resulted solely from the late-gestation exposure to viral mimetic poly(I:C). Using a genome-wide approach, Richetto et al. [59] then provided evidence that DNA methylation-dependent mechanisms may underlie the long-term consequences of immune activation on brain function. Prenatal immune activation resulted in genome-wide DNA methylation changes in the adult’s prefrontal cortex; these changes were largely dependent on the timing of exposure, consistent with the time-specific behavioral effects. Among 1408 genes and 1756 genes that were found to be differentially methylated in the mid-gestation and late-gestation time points, respectively, only 167 genes were common to both exposures. The DNA methylation changes induced at both time points were enriched at genes involved in neuronal differentiation and included neuregulin 1 (*Nrgn1*), neurexin 2 (*Nrxn2*), and neuronal differentiation 6 (*Neurod6*). On the other hand, differentially methylated regions following the GD9 immune-activation were primarily associated with genes involved in Wnt signalling, whereas the GD17 immune activation was primarily linked to epigenetic changes in genes involved in the differentiation of the GABAergic neuronal system. Importantly, for a subset of genes, the authors confirmed that the DNA methylation differences correlated with changes in gene expression, further suggesting that the altered methylation levels were functionally relevant. It is important to note that the genes differentially methylated in the adult PFC were not differentially methylated in the PFC at postnatal day (PD) 1 of the prenatally immune-challenged animals. This finding provides additional evidence that, rather than inducing permanent effects on specific CpG sites, an early-life insult may affect the developmental programming of the machinery that regulates DNA methylation throughout life, allowing the DNA methylation status of genes to be regulated in an age- and state-dependent manner.

### 3.4. Maternal Exposure to Drugs

Maternal exposure to drugs, both therapeutic and recreational, has been linked to neurobehavioral consequences for the offspring, and the possible mediating role of epigenetic mechanisms has been proposed [82]. The effects of drugs of abuse, such as cocaine, on the adult brain epigenome are well-established [83]; however, the link between maternal drug exposure and epigenetic changes in the brain of offspring is just emerging.

#### 3.4.1. Cannabis

An interesting study explored the role of epigenetic mechanisms, specifically histone modifications H3K4me3 and H3K9me2, in the neurodevelopmental effects of Δ-9-tetrahydrocannabinol (THC), the main active component of marijuana or cannabis [50]. Maternal cannabis use during pregnancy has been associated with lasting consequences for the offspring, including an increased risk of developing drug addiction and neuropsychiatric disorders [84,85]. Lasting effects of THC on the dopaminergic brain’s reward pathway, projecting from the ventral tegmental area to the nucleus accumbens (NAc), provided a good candidate for the study, as this pathway has been strongly implicated in drug abuse. In particular, the expression of the dopamine D2 receptor (*DRD2*) has been shown to be reduced in adult drug addicts [86]. In addition, the examination of human fetal tissue from pregnancies where the mothers used cannabis demonstrated that in utero cannabis exposure is associated with reduced *DRD2* gene expression in the fetal NAc at approximately 20 weeks of gestation [50].

DiNieri et al. [50] used a rat model to examine whether prenatal exposure to cannabis, or more specifically to THC, may induce the disruption of *Drd2* gene regulation in the NAc that persists into adulthood, and whether epigenetic mechanisms are involved in this effect. Importantly, gestational exposure to THC led to reduced *Drd2* gene expression in the offspring’s NAc, both at PD2 (corresponding to gestational week 20 in humans) and in adulthood (PD62). Decreased *Drd2* expression in adult NAc was associated with the following epigenetic changes: a decreased histone mark H3K4me3 typically associated with active promoters and an increased H3K9me2 typically associated with a repressed chromatin state; changes in these two marks were further associated with a decreased binding of the RNA polymerase II at the *Drd2* gene promoter. Moreover, the adult offspring prenatally exposed to THC also exhibited a reduced number of D2 receptors in the NAc and an increased sensitivity to opiate reward. In summary, this study provides evidence that prenatal cannabis exposure can alter the developmental epigenetic programming of *D2DR* expression within the brain’s reward pathway, resulting in a lasting reduction of D2 receptors that may contribute to addiction vulnerability. Interestingly, human genetic studies have also linked genetic polymorphisms in the *D2DR* gene to addiction [87], implying that genetic and environmentally-induced epigenetic factors may interact to bring about changes in *D2DR* gene expression that may promote vulnerability to addiction.

#### 3.4.2. Cocaine

Another study explored maternal cocaine exposure during the second and third gestational week and the associated epigenetic consequences for the offspring [57]. Maternal cocaine exposure resulted in changes in the expression of epigenetic regulators, DNMTs, and global DNA methylation changes in the hippocampal neurons of the male neonatal and prepubertal offspring. Several genes involved in cellular differentiation and survival as well as in synaptic plasticity were shown to be hypermethylated or hypomethylated, and this was associated with altered gene expression. However, in this study, cocaine-induced molecular changes were not linked to phenotypic differences such as changes in the hippocampal tissue and behavior, and further studies are required to provide the link between maternal cocaine exposure, epigenetic mechanisms, and the disruption of brain function and behavior in offspring.

#### 3.4.3. Ethanol

The effects of maternal exposure to alcohol on the offspring’s epigenome and brain structure have also been explored [52]. High levels of alcohol consumption in pregnant women can result in fetal alcohol syndrome, structural brain abnormalities, and later behavioral problems in the offspring. This study used the *A^vy^* allele as an epigenetic biosensor: A^vy^ mice (the *A^vy^*/*a* genotype) contain a meta-stable, DNA methylation-sensitive *A^vy^* allele in the *Agouti* gene locus, which determines the coat color. DNA methylation levels at CpG sites within *A^vy^* are established during early embryogenesis and are probabilistic events, resulting in a wide distribution of the coat color of *A^vy^* mice, ranging from pure yellow (hypomethylation of the *A^vy^*) to pseudoagouti brown (hypermethylation of the *A^vy^*). Kaminen-Ahola et al. [52] demonstrated that maternal exposure to ethanol from GD0.5 to GD8.5 increased the probability of hypermethylation and silencing at this locus and more mice with an agouti-colored coat. Importantly, the congenic a/a siblings of the A^vy^ mice exhibited postnatal growth restriction and changes in craniofacial morphology, similar to fetal alcohol syndrome in humans, implying that the expression of genes other than *A^vy^* was also affected by prenatal ethanol exposure. This study provides evidence that the epigenome is vulnerable to ethanol during early development and suggests that epigenetic mechanisms may contribute to ethanol-induced neurodevelopmental effects.

In summary, several studies show that prenatal exposure to recreational drugs, such as marijuana, cocaine, and alcohol, may induce lasting epigenetic changes in the offspring which, in turn, may contribute to behavioral alterations and increased psychiatric risk in the prenatally-exposed offspring.

## 4. Evidence from Human Studies

In humans, it is very challenging to establish the epigenetic link between prenatal environmental exposures and behavioral/psychiatric disorders occurring later in life. First, humans are always exposed to a mixture of environmental agents and it is difficult to clearly dissociate the contribution of a single factor to any neurodevelopmental outcome. Second, the brain as a target tissue is inaccessible in living humans and hence, for now, we have to rely on epigenomic studies of peripheral tissues. It is fortunate that increasing evidence shows that the epigenetic profiling of peripheral tissues such as blood or buccal cells may be informative for behavioral outcomes [48,88]. In this review, we will focus on a few human studies which linked prenatal environmental risk factors and epigenetic changes of relevant neuronal genes in human peripheral tissues. We are hopeful, though, that at some point in the future, carefully designed longitudinal birth cohorts will become available, with rich information on the individual exposomes, psychiatric clinical data, and comprehensive tissue collections (including postmortem brains) for epigenetic analyses [89,90]. These studies would allow for the analyses of epigenetic changes over time and would provide the opportunity to relate epigenetic changes to specific environmental exposures as well as to the development of psychiatric disorders.

### 4.1. Dutch Famine Study

The first experimental evidence that in utero environmental conditions can cause long-lasting epigenetic changes in humans was provided by the famed Dutch Famine Study. This is a seminal epidemiological study that established important associations between the prenatal environment and long-term mental health outcomes [13,91]. The study is based on a “natural experiment” involving a severe famine that occurred during World War II, from October 1944 to May 1945, during the Nazi blockade of the occupied Western Holland. The famine achieved its peak from March to April 1945, when the population was nutritionally depleted with daily food rations of as little as 500–1000 kcal. From the data collected over the decades following the famine, this study made associations between maternal periconceptional exposure at the famine’s peak and an increased risk of the following neurodevelopmental outcomes in offspring: (i) neural tube defects; (ii) diagnoses of schizoid personality disorder at age 18; and (iii) SCZ in adulthood [21].

In the case of the Dutch Famine study, at least two risk factors may have interacted to affect neurodevelopment in the offspring: (i) malnutrition due to the famine; and (ii) maternal stress due to the famine, war, and many other stressful events associated with this tragic life situation. Regarding the increased risk of SCZ, the role of nutritional deprivation is likely to be prevailing [92], and among many candidate nutrients, a lack of folic acid has been proposed as a major factor affecting neurodevelopment [92]. Folic acid is critical for normal brain development and maternal folate supplementation during the periconceptional period has been shown to decrease the risk of neurodevelopmental disorders in children, including neural tube defects, severe language delays, autism, and cognitive impairments; outcomes that could be antecedents of SCZ [21,93,94]. Folate is also an important epigenetic factor; it is used for the production of methyl donors and is required for DNA methylation. This provided a plausible hypothesis that nutritional (or folate) deficiency could affect neurodevelopment and a later risk of SCZ, at least in part, via epigenetic mechanisms.

Importantly, periconceptional exposure to famine was associated with widespread DNA methylation changes in the blood, when the individuals prenatally exposed to famine were compared to their unexposed, same-sex siblings at approximately 60 years of age [95,96]. The affected genes included the insulin-like growth factor 2 (IGF2), an imprinted gene that plays an important role in growth and development [95], as well as additional genes implicated in growth and metabolic pathways [96]. Together, these studies show that periconceptional maternal exposure to famine can have a lasting effect on the offspring’s DNA methylome, with potential consequences for brain structure and function. Importantly, many psychiatric disorders, including SCZ [97], show widespread epigenetic changes not only in specific brain regions but also in peripheral blood [98], further supporting the hypothesis that prenatal environmental exposures can contribute to the development of psychopathology by inducing long-lasting changes in the epigenome.

### 4.2. Prenatal Stress and Maternal Depression

Several human studies provided a link between prenatal maternal stress or maternal depression and epigenetic changes within the gene encoding glucocorticoid receptor (*NR3C1*), which is associated with the HPA axis. For instance, an analysis of cord blood samples from children born to mothers suffering from depression during the third semester of pregnancy demonstrated increased DNA methylation of the *NR3C1* gene, when compared to controls [99]. Levels of *N3RC1* methylation in fetal cord blood were further found to predict infant HPA reactivity (cortisol response to stress) at three months of age, suggesting a possible functional consequence of this epigenetic variation. In another study, maternal depressive symptoms during pregnancy predicted not only increased *NR3C1* DNA methylation in buccal cells of male infants but also decreased *BDNF IV* DNA methylation in both male and female infants [100], further implicating epigenetic changes in *BDNF* in response to maternal adversity, as previously shown by animal studies [51,61,62]. Maternal stress during pregnancy was also shown to affect offspring’s *N3RC1 DNA* methylation beyond infancy [101]. Maternal exposure to intimate partner violence during pregnancy was associated with increased *N3RC1 DNA* methylation levels in the whole blood samples of 10–19 year-old offspring. In these studies [99,101], epigenetic changes were present in the offspring’s blood samples, but not in maternal blood samples, suggesting that stress-induced epigenetic dysregulation of the *N3RC1* gene occurs during developmental epigenetic programming. Together with the animal studies, these findings further support the hypothesis that maternal stress during pregnancy can alter in utero epigenetic programming, contributing to neurodevelopmental and behavioral deficits in the offspring.

### 4.3. Toxicological Exposures

Finally, human studies have also explored epigenetic changes in the offspring in response to maternal exposure to various toxicants during pregnancy. For instance, we have shown that maternal exposure to high levels of the endocrine disruptor BPA is associated with increased *BDNF* DNA methylation levels in male offspring, which was detectable in cord blood at birth. Importantly, behavioral analysis of the same cohort, conducted on children at 3–5 years of age, showed that high maternal BPA exposure is associated with disturbed emotional regulation and increased aggressive behavior in boys, whereas girls were not significantly affected [71], further implicating a possible role of epigenetic mechanisms in BPA-induced, sex-specific behavioral effects. Maternal smoking during pregnancy was also shown to be associated with epigenetic modifications within the *BDNF* gene, but in adolescent offspring, further confirming that the induced epigenetic changes can be long-lasting [102]. While early exposures to toxicants were rarely linked to specific psychiatric disorders, possibly because this has not been sufficiently studied, the epigenetic dysregulation of the *BDNF* gene may well represent one of the mediators of psychiatric risk shared by many different environmental exposures, as seen in both the animal and human studies explored in this review. In addition to *BDNF*, many other genes are shown to be epigenetically dysregulated and could possibly contribute to neurodevelopmental effects induced by common toxicants such as air pollutants [103], and additional studies in this area are warranted.

## 5. Future Directions and Challenges

The experimental evidence summarized above strongly suggests that epigenetic mechanisms may underlie the effects of prenatal environmental risk factors on long-term brain function and psychiatric risk. While substantial experimental evidence is still lacking, this field holds great promise for improving our understanding of the pathophysiology of mental disorders, and may initiate the development of novel diagnostic and preventive approaches in psychiatry. However, the field faces many challenges and here we will briefly discuss some future directions and areas that need to be addressed in future studies.

### 5.1. Dose and Developmental Window of Exposure

The studies presented here clearly show that the effects of environmental exposures may significantly depend on the dose/level and the gestational timing of exposure, and it will be important to incorporate these variables into the design of future studies.

### 5.2. Sex-Specific Effects

One of the important things to emphasize is that the epigenetic and behavioral effects of prenatal environmental exposures are often found to be sex-specific (Table 1). This is not surprising considering that the brain epigenome and epigenetic regulation in the brain are reportedly found to be sex-dependent, and many mental disorders show sex bias in terms of both prevalence and disease severity [53,104]. However, similar to other areas in neuroscience, many studies of prenatal exposures on brain function have so far focused on male subjects (Table 1). Therefore, it will be very important to include both sexes in all future studies, as these findings can significantly improve our understanding of the mechanisms that are responsible for sex differences in neurodevelopmental disorders, hopefully leading us to better targeted treatments and interventions.

### 5.3. Peripheral Biomarkers

As previously mentioned, one of the major challenges in studying the effects of environmental exposures in humans is the inaccessibility of the target brain tissue in living subjects. We have previously shown that BPA-induced epigenetic changes in peripheral blood are indicative of epigenetic changes in the brain and of behavioral phenotypes, and we have translated these findings to humans [48]. However, it will be important to further study correlations between the blood and brain epigenome in response to environmental exposures, as these results will help us understand the relevance of epigenetic changes in peripheral tissues for predicting neurodevelopmental outcomes in humans.

### 5.4. Technological Advancements and Integrative Approaches

Future studies, both in animals and particularly in the tissues of human subjects, will require the implementation of more advanced epigenomic techniques, which have been rarely used so far. Technologies that are based on next generation sequencing, such as whole-genome bisulfite sequencing (for DNA methylation analysis), ChIP-seq (for histone modification analysis), and ATAC-seq (for the analysis of chromatin accessibility and organization), provide a better resolution and coverage, and allow for more accurate profiling of epigenetic modifications at the level of the whole genome. In addition to this, a cell-type specific epigenomic profiling [105] in the brain and peripheral tissues, and a distinction between DNA methylation and hydroxymethylation, will be very beneficial in our efforts to better understand how epigenetic mechanisms and epigenetic variation contribute to prenatally-induced neurodevelopmental disorders. Finally, as epigenetic changes are only one class of molecular changes contributing to brain disorders, it will be very important to integrate epigenomic data with gene expression data and genetic information, in order to understand the functional role of developmentally-induced epigenetic changes and to place them in a wider context of gene (dys)regulation in neurodevelopmental disorders.

In summary, a global research effort combining well-designed human and animal studies, with comprehensive epigenomic analyses of peripheral and brain tissues over time, will be necessary to improve our understanding of the epigenetic basis of neurodevelopmental disorders; an effort which we believe will be very rewarding.

## Figures and Tables

**Table 1:** Animal studies providing an epigenetic link between prenatal environmental exposure and neurobehavioral outcomes.

Study	Species (Strain)	Stress Paradigm	Offspring Sex and Age	Brain Area	Analysis	Gene (Epigenetic Changes)	Epigenetic Regulators	Neurobehavioral Outcome	Sex Specificity
Basil et al., 2014 [49]	Mouse (C57BL/6N)	Maternal immune activation (GD17)	Males and females, PD42	Hy	Sequenom EpiTYPER assay	Mecp2, LINE1	-	-	Males and females; more profound changes in females
DiNieri et al., 2011 [50]	Rat (Long Evans)	THC rat model (GD5-PD2)	Males, PD62	NAc	ChIP (H3K9me2, H3K4me3, RNA polymerase II)	Drd2	-	Increased sensitivity to opiate reward; Drd2 transcriptional changes	-
Dong et al., 2015 [51]	Mouse (Swiss albino ND4)	Restrain stress (GD7-GD21)	Males, PD75	FC, Hy	MeDIP, hMeDIP, ChIP	Bdnf	Dnmt1, Tet1	Hyperactivity; Impaired social interaction; Bdnf transcriptional changes	-
Kaminen-Ahola et al., 2010 [52]	Mouse (C57BL/6J Agouti^vy^)	Ethanol exposure (GD0.5-GD8.5)	Males and females, PD28–30	FB	Bisulfite Sequencing, Gene Expression Arrays	IAPs	-	Fetal alcohol syndrome-like features	-
Kundakovic et al., 2013 [53]	Mouse (BALB/c)	BPA exposure (GD0-GD19)	Males and females, PD30–70	PFC, Hi, Hy	Bisulfite Pyrosequencing	Esr1	Dnmt1, Dnmt3a	Disrupted exploratory, social and anxiety-like behavior; transcriptional changes in Esr1, Esr2 and Esrrg	Sex specific changes
Kundakovic et al., 2015 [48]	Mouse (BALB/c)	BPA exposure (GD0-GD19)	Males and females, PD28 and PD60	Hi	Bisulfite Pyrosequencing	Bdnf IV, Bdnf IX, Grin2b	Dnmt1, Gadd45b	Disrupted exploration of a novel object; Bdnf and Grin2b transcriptional changes	Changes were observed in males
Labouesse et al., 2015 [54]	Mouse (C57BL/6N)	Maternal immune activation (GD17)	PD80–100	mPFC	MeDIP, hMeDIP, ChIP (MeCP2)	Gad1, Gad2	MeCP2	Impaired working memory and social interaction deficits; Gad1 and Gad2 transcriptional changes	-
Mueller and Bale, 2008 [55]	Mouse (C57BL/6N:129)	Chronic, variable stress during early, mid and late gestation	Males and females, 6–16 weeks	Hi, Am	Bisulfite Pyrosequencing	Crf, Nr3c1	-	Maladaptive behavioral stress-responsivity, anhedonia, and an increased sensitivity to SSRI treatment in males	Sex specific changes
Matrisciano et al., 2013 [56]	Mouse (Swiss albino ND4)	Restrain stress (GD7-GD21)	Males, PD60	PFC, Hi	MeDIP, hMeDIP, ChIP (Dnmt1, MeCP2)	Reelin, Gad1	Dnmt 1, Dnmt 3a	Hyperactivity; Impaired social interaction, prepulse inhibition, and fear conditioning; Reelin and Gad1 transcriptional changes	
Novikova et al., 2008 [57]	Mouse (CD1)	Cocaine exposure (GD8-GD19)	Males, PD3 and PD30	Hi	Bisulfite sequencing, MeDIP/CGI array	Genome-wide	Dnmt1, Dnmt 3a	Transcriptional changes in Gpr73, Plk2, Prpn5, Mapk1, Impa1, Pyrk3, Gata4, Mtap6, Gtf3c1, Coq7	-
Onishchenko et al., 2008 [58]	Mouse (C57BL/6/Bkl)	MeHg exposure (GD7-PD7)	Males, 9 weeks	DG	ChIP (H3ac, H3K27me3), Ms-SNuPE	Bdnf		Depresion like behavior; transcriptional changes in Bdnf	-
Richetto et al., 2017 [59]	Mouse (C57BL6/N)	Maternal immune activation (GD9 and GD17)	Males, PD100	mPFC	SureSelectXT capture sequencing assay and EpiTYPER	Genome wide (Dlx1, Lhx5, Lhx8, Wnt3, Wnt8a, Wnt7b, Efnb3, Mid1, Nlgn1, Nrxn2, Nf2, etc.)	-	Impaired sensorimotor gating, social interaction and spatial memory; transcriptional changes in Dlx1, Wnt3, Mid1, Nlgn1, Nf2	-
Schraut et al., 2014 [60]	Mouse (C57BL6/J, 5-Htt +/+ and 5-Htt +/−)	Restrain stress (GD13-GD17)	Females, PD95	Hi	MeDIP-on-Chip	Genome wide	-	Anxiety-related behavior; Mdb transcriptional changes	-
St-Cyr S and McGowan, 2015 [61]	Mouse (C57BL/6N)	Exposure to predator odor (GD11-GD18)	Females and males, PD90	Hi, Am	Bisulfite Pyrosequencing	Bdnf	-	Increased avoidance and decreased predator-odor associated activity; Bdnf and Crhr1 transcriptional changes	Males and females, but females showed a greater increase in CORT level
Zheng et al., 2016 [62]	Mouse (Kunming)	Restrain stress (GD5-delivery)	Males, PD40	Hi	MeDIP,ChiP (H3K14ac)	Bdnf	Dnmt1, Hdac1, Hdac2,	Depressive-like and anxiety-like behaviors, Bdnf transcriptional changes	-

Abbreviation List: GD—gestational day, PD—postnatal day, THC—delta-9-tetrahydrocannabinol, BPA—Bisphenol A, MeHg—methylmercury, Hy—hypothalamus, NAc—nucleus accumbens, FC—frontal cortex, FB—forebrain, PFC—prefrontal cortex, mPFC—medial prefrontal cortex, Hi—hippocampus, Am—amygdala, DG—dentate gyrus, ChIP—Chromatin Immunoprecipitation, MeDIP—Methylated DNA immunoprecipitation, hMeDIP—Hydroxymethylated DNA Immunoprecipitation, MeDIP-CGI-arrays—methylated DNA immunoprecipitation coupled with CpG island microarrays, Ms-SNuPE—Methylation-sensitive single-nucleotide primer extension, LINE1—long interspersed element, IAPs—intracisternal A-particles.
